# Congenital Uterovaginal Prolapse in a Newborn

**DOI:** 10.1155/2018/1425953

**Published:** 2018-06-27

**Authors:** Zenebe Wolde Jijo, Million Teshome Betele, Abdurazak Shemolo Ali

**Affiliations:** Department of Obstetrics and Gynecology, College of Medicine and Health Sciences, Hawassa University, Hawassa, Ethiopia

## Abstract

**Background:**

Uterovaginal prolapse is a rare condition in newborns which is usually associated with spinal cord defects. It is usually diagnosed at birth. Different treatment options have been proposed for genital prolapse in newborns. Most of the treatment options recommended are conservative; radical approach is rarely suggested for the treatment of these cases.

**Case Details:**

We report a 6-hour-old newborn that was diagnosed to have congenital uterovaginal prolapse and was successfully managed conservatively with digital reduction of the mass and strapping of both legs together.

**Conclusion:**

Congenital uterovaginal prolapse in newborns can be managed conservatively with simple digital reduction followed by strapping of the legs together; this approach is simple and cost-effective and has no associated complications.

## 1. Introduction

Congenital uterovaginal prolapse in a newborn is a rare condition which is usually diagnosed at birth or in the first few days of life. Often times the condition is associated with spinal cord defects [1–3]. Various management options have been suggested by different authors, which include simple digital reduction, use of pessary, use of Foley catheter, and other self-retaining devises [3–6]. We are reporting a case of neonatal uterovaginal prolapse which was diagnosed at birth related to myelomeningocele and was successfully managed by simple digital reduction followed by application of bandage straps over both thighs and buttocks.

## 2. Case Description

A 6-hour-old female neonate born from a 22-year-old Para 1 mother after term pregnancy. The newborn had protrusion of mass per vagina since birth; she also has a defect at the lower back with no discharge since the time of birth. Additionally the mother has noticed deformity on both legs and feet which barely move. The baby was active since the time of delivery and is sucking well and she passed meconium. The mother had two ANC visits and was vaccinated according to the national schedule and she reported the whole course of the pregnancy as uneventful. She did not have any known medical illness and has never taken any medication during the entire course of the index pregnancy except iron folate which was prescribed during the ANC visit.

She had spontaneous onset of labor and the membrane was ruptured spontaneously intrapartum. She gave birth to a 2330-gram female neonate after 5 hours of labor at a local health center. Baby had good APGAR score. Health care providers at the health center noticed defect at the back of the baby and referred her to Hawassa University Comprehensive Specialized Hospital (HUCSH) with the diagnosis of spinal bifida.

**Figure 1:**
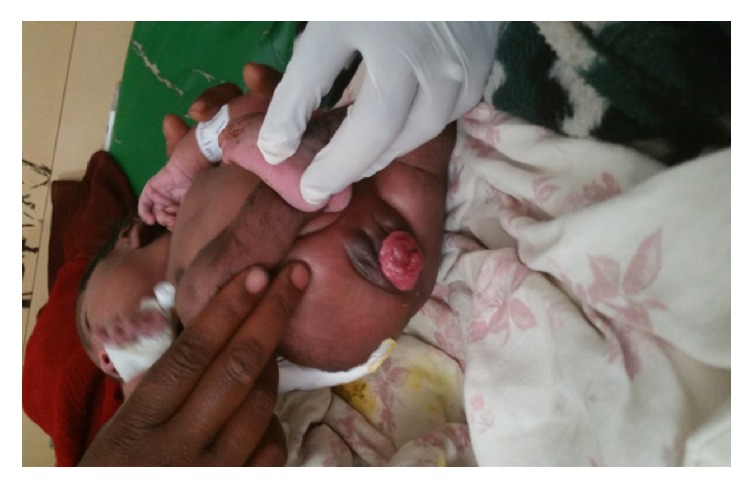
Picture of the uterovaginal prolapse.

**Figure 2:**
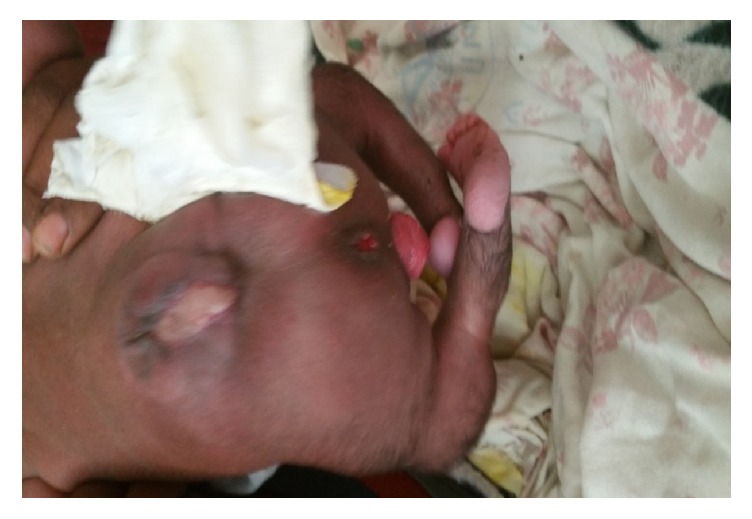
Picture of the spinal cord defect, uterovaginal prolapse, and club feet.

During the initial evaluation at HUCSH the baby was active, vital signs were in the normal limits, and all neonatal reflexes were intact. There was 4x4 cm pink mass protruding through the introitus, cervical os is noted at the tip of the mass, the external genitalia appears normal, no discharge or bleeding from the mass, and the mass was reducible digitally and increases in size when the baby cries (see Figure 1). There was also 4x4 cm defect at the lumbosacral region the major portion of which is covered with skin while the lower edge is open, no discharge from the mass (see Figure 2). Additionally the newborn had bilateral club foot deformity (see Figure 2).

On investigation, the complete blood count was normal, creatinine level was 0.4mg/dl, and transfontanelle ultrasound scan shows mild dilatation of the lateral and third ventricles with an index of “mild hydrocephalus” and abdominal ultrasound was normal.

**Figure 3:**
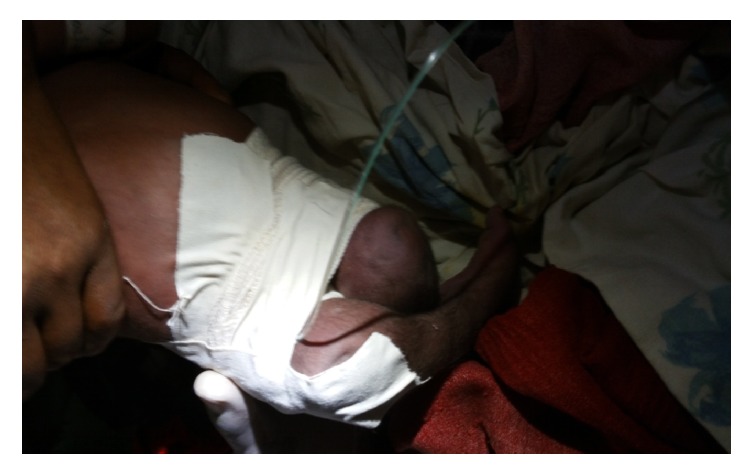
Picture of the newborn with Foley catheter in place and legs strapped.

**Figure 4:**
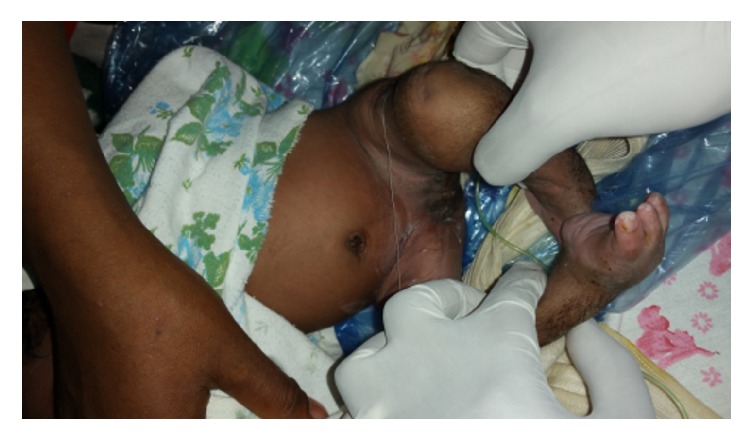
Picture of the newborn after successful reduction of the prolapse.

After obtaining consent from the parents, under aseptic technique, the baby was catheterized, the vaginal mass was reduced digitally, and bandage was applied from the lower abdomen, both buttocks and legs were strapped in the bandage to the level of the mid-thigh leaving an opening at the anal orifice for passage of stool (see Figure 3). The bandage was removed after 72 hours at which time the mass was completely reduced and there was no recurrence of the prolapse afterwards (see Figure 4). Unfortunately one day after the removal of the bandage the baby started to shoot fever and had difficulty of sucking, on examination she had tachycardia and tachypnea and was febrile, she had depressed reflexes, the lower border of the meningocele got ruptured, her fontanels were bulged, and she was diagnosed with ruptured myelomeningocele and meningitis. The plan was to start her on antimeningitis drugs and repair the spinal cord defect after improvement but the parents insisted and went home against medical advice frustrated by her multiple anomalies. We communicated with the parents after her discharge and were informed that the baby died 7 days after she went home, but there was no recurrence of the prolapse throughout her stay.

## 3. Discussion

Uterovaginal prolapse is defined as the downward displacement of the normal pelvic structures to or through the vagina and introitus. The first case of genital prolapse in a newborn was reported in 1723. The first formal review on the condition was written by Ballantyne and Thomson in 1897. Neonatal uterovaginal prolapse is generally a rare condition [7, 8].

The uterus and the vagina are suspended in their normal anatomic place by the pelvic diaphragm and the endopelvic fascia (which is composed of uterosacral ligaments, cardinal ligaments, and the pubocervical fascia). Congenital uterovaginal prolapse is a result of weakness in the pelvic muscle and ligaments; this weakness could be either secondary to congenital weakness in the pelvic musculature or defective innervation [5, 9, 10]. The pelvic muscle support is normally innervated by the perineal branch of the sacral nerve. Lesions to these nerves or their spinal cord component results in paralysis of the pelvic diaphragm with subsequent descent of the pelvic visceral structures [9].

Even though the exact etiology of uterovaginal prolapse in newborns is not well known, few risk factors have attributed to the development of the condition in newborns, the most common of which is spinal bifida (82-86%) [1, 3, 5, 9]. Our case is also a patient with congenital uterovaginal prolapse which is associated with spinal bifida at the lumbosacral region. Prolonged breech presentation, birth trauma, congenital cutis laxa, and prematurity are also among the other risk factors related to the condition [5, 11].

Timely management of uterovaginal prolapse in newborns is essential to prevent injury and metaplasia to the endometrium from prolonged exposure to the external environment and to prevent other associated complications like urinary retention and obstructive uropathy [5, 9].

Based on few available cases reported so far, authors recommend conservative treatment of uterovaginal prolapse in newborns as effective management options. After reduction of the prolapse, since the maternal estrogen exposure ceases and edema subsides the uterus will be fixed to its normal anatomic location. The reported success rate with conservative management is more than 90%. The major concern with conservative management of UVP is recurrence of the condition [5, 9].

Use of vaginal pessary after digital reduction has been reported as successful intervention which involves digital reduction of the prolapsed mass followed by insertion of pessary and fixing the pessary to the vaginal wall using fine stitches, the pessary is then removed after one month [6]. Other authors reported successful reduction of the prolapse using purse string suturing technique which involves digital reduction of the prolapse followed by application of purse string suture over the labia to reduce recurrence, reports indicate success in reduction of the prolapse 6 days following repair of the spinal cord defect [4]. Additionally another case report showed successful reduction of genital prolapse in a newborn after employing manual reduction under general anesthesia and partial fusion of the labia majora by using intermittent suturing [12]. Similar success was reported in a case who was managed with insertion of Foley catheter after digital reduction the prolapsed mass [3]. Our case was successfully managed with digital reduction of the mass and strapping of both buttocks and lower extremities together for three days; there was no recurrence of the condition after removal of the bandages.

Rarely radical approaches especially for cases with associated spinal cord defect and recurrent prolapse following conservative management have been suggested in literatures. These approaches are recommended when there is repeated prolapse despite multiple reduction of the prolapse or when there is evidence of vaginal hypertrophy or laceration. Methods of fixation that have been suggested include sling procedure, cervical sacropexy, ventrosuspension, and abdominal sacrocolpopexy. There were also rare cases which were reported to be managed by hysterectomy and cervical amputation [5, 12].

## 4. Conclusion

Uterovaginal prolapse is a rare condition in newborn babies which is usually associated with spinal bifida. The condition can be managed successfully with digital reduction and strapping of both extremities together in a gauze bandage. We recommend this simple and effective management for cases of congenital uterovaginal prolapse in newborns as it is easy to apply and cost-effective, which can be performed at a primary facility and has no associated complications other than possible recurrence of the prolapse.
